# Association of Fine Particulate Matter and Residential Greenness With Risk of Pulmonary Tuberculosis Retreatment: Population-Based Retrospective Study

**DOI:** 10.2196/50244

**Published:** 2024-08-12

**Authors:** Tonglei Guo, Fei Shen, Henan Xin, Jiang Du, Xuefang Cao, Boxuan Feng, Yijun He, Lingyu Shen, Yuanzhi Di, Yanxiao Chen, Zihan Li, Qi Jin, Hongzhi Li, Chunming Zhang, Lei Gao

**Affiliations:** ^1^NHC Key Laboratory of Systems Biology of Pathogens, National Institute of Pathogen Biology, Chinese Academy of Medical Sciences & Peking Union Medical College, Beijing, China; 2 National Institute of Pathogen Biology and Center for Tuberculosis Research, Chinese Academy of Medical Sciences & Peking Union Medical College, Beijing, China; 3 Department of Tuberculosis Prevention and Control, The Sixth People’s Hospital of Zhengzhou, Zhengzhou, China; 4 College of Public Health, Zhengzhou University, Zhengzhou, China; 5 Department of Pathogen Biology, Hainan Medical University, Haikou, China

**Keywords:** tuberculosis, PM2.5, particulate matter, air pollution, greenness, retrospective study, pulmonary, retreatment

## Abstract

**Background:**

The evidence on the association of fine particulate matter with an aerodynamic diameter of 2.5 μm or less (PM_2.5_) with pulmonary tuberculosis (PTB) retreatment is limited. There are no data on whether greenness exposure protects air pollution–related PTB retreatment in patients with prior PTB.

**Objective:**

In a population-based retrospective study, we aimed to investigate the influence of PM_2.5_ and residential greenness on the risk of PTB retreatment.

**Methods:**

A total of 26,482 patients with incident PTB, registered in a mandatory web-based reporting system between 2012 and 2019 in Zhengzhou, China, were included in the analysis. The exposure to PM_2.5_ was assessed based on the China High Air Pollutants dataset, and the level of greenness was estimated using the Normalized Difference Vegetation Index (NDVI) values. The associations of PTB retreatment with exposure to PM_2.5_ and greenness were evaluated, respectively, considering the local socioeconomic level indicated by the nighttime light index.

**Results:**

Among the 26,482 patients (mean age 46.86, SD 19.52 years) with a median follow-up time of 1523 days per patient, 1542 (5.82%) PTB retreatments were observed between 2012 and 2019. Exposure to PM_2.5_ was observed to be significantly associated with the increased risk of PTB retreatment in fully adjusted models with a hazard ratio of 1.97 (95% CI 1.34‐2.83) per 10 μg/m^3^ increase in PM_2.5_. Patients living in the regions with relatively high quartiles of NDVI values had a 45% lower risk of PTB retreatment than those living in the regions with the lowest quartile for the 500 m buffers (hazard ratio 0.55, 95% CI 0.40‐0.77). Such a protective effect of residential greenness was more pronounced among patients living in lower nighttime light areas. The strength of the association between PM_2.5_ exposure and the risk of PTB retreatment was attenuated by greenness. No significant association was observed between NDVI and the incidence of drug resistance.

**Conclusions:**

Long-term exposure to PM_2.5_ might be a risk factor for PTB retreatment, while an increased level of residential greenness was found to be associated with reduced risks of PTB retreatment. Our results suggest strengthening the control of ambient air pollution and improving residential greenness may contribute to the reduction of PTB retreatment.

## Introduction

Tuberculosis (TB) caused by the infection of *Mycobacterium tuberculosis* (MTB) is a major infectious disease and caused 1.6 million deaths worldwide in 2021 [1]. More crucially, eight countries accounted for more than two-thirds of all estimated incident cases worldwide, among which China is the country with the third highest burden of TB [1]. TB recurrence refers to a second episode of TB that occurs after the first episode has been considered cured, which is caused by reinfection (exogenous infection with a new strain) or relapse (an endogenous reactivation of the same strain of MTB, mostly due to inadequate treatment and drug resistance) [2]. Patients who complete TB treatment in areas with a high prevalence of TB face a considerable risk of TB retreatment which is an important obstacle for the End TB strategy [1,2].

In recent years, air pollution has been a major public health challenge in the world [3]. Evidence for the association between fine particulate matter with an aerodynamic diameter of 2.5 μm or less (PM_2.5_) and PTB retreatment is limited. Recent studies have suggested that short- and long-term exposures to outdoor PM_2.5_, which may inhibit cellular immunity to MTB, were significantly associated with the risk of PTB [4,5]. In addition, previous studies suggest that exposure to greenness is associated with improved health outcomes, such as better mental health status [6], more physical activity, better weight management [7], healthier sleep durations [8], better cardiovascular status [9], and increased longevity [10]. Several potential mechanisms may explain these associations, including reduction of air pollutants, noise, and heat; encouraging healthy physical activity; and recovery of physiological stress [11]. Zhu et al [12] reported that long-term exposure to PM_2.5_ was positively associated with both pulmonary tuberculosis (PTB) or smear-positive pulmonary tuberculosis (SPPTB) incidences in China; meanwhile, the Normalized Difference Vegetation Index (NDVI) has attenuated the association between PM_2.5_ and SPPTB incidence. Additionally, in a Chinese cohort study of 1621 patients with multidrug-resistant tuberculosis (MDR-TB) treatment, patients with higher greenness exposure levels were associated with decreased risk of all-cause mortality among patients living in lower nighttime light (NTL) areas [13]. However, there is no evidence of the impact of residential greenness on the risk of PTB retreatment and drug resistance. Besides, it is completely unknown whether greenness exposure protects air pollution–related PTB retreatment in patients.

Therefore, a population-based retrospective study was conducted to examine the association between the risk of PTB retreatment and exposure to PM_2.5_ as well as greenness exposure in Zhengzhou city in China. Furthermore, the effect modification of greenness on PM_2.5_ and PTB retreatment was investigated. The findings of this study might provide evidence for strengthening PTB prevention and control from the perspective of public health.

## Methods

### Study Overview

This retrospective study, addressing the association of PTB retreatment with exposure to PM_2.5_ and greenness, was conducted in Zhengzhou, which is the capital city of Henan Province in China, with a resident population of approximately 12.83 million in 2022 and an area of 7567 km^2^. The data of PTB cases registered in the Tuberculosis Information Management System, a mandatory we-based reporting system, during the 2012‐2019 period in Zhengzhou were exported for the current analysis. The estimates of greenness were based on NDVI, a measure derived from the Moderate-Resolution Imaging Spectroradiometer of the National Aeronautics and Space Administration’s Terra Satellite images and a metric widely used in previous epidemiological studies for quantifying outdoor greenness [13,14]. China High Air Pollutants (CHAP) using a mature machine learning-based method (an enhanced space-time extremely randomized trees) was used to estimate the annual mean concentrations of ambient PM_2.5_ with a high-resolution (1 km). The CHAP dataset has been widely used in previous epidemiological studies [15,16].

### Study Population

All registered PTB cases in Tuberculosis Information Management System in Zhengzhou between January 1, 2012, and December 31, 2019, were included for analysis. Each included patient contained information on age, gender, occupation, current address, history of PTB, original residence, type of TB, date of PTB symptom report, drug-resistant results, date of diagnosis, and results of smear microscopy or culture. Follow-up for patients with PTB was conducted through the mandatory web-based reporting system. Patients who lacked results of smear microscopy or culture during follow-up or migrant patients who moved out of Zhengzhou were excluded because information, including current address and date of retreatment diagnosis, on these patients was not available. To avoid privacy leakage and confidentiality issues, patients’ names and resident ID numbers were excluded. Inclusion criteria were as follows: patients registered in a health facility in Zhengzhou between January 1, 2012, and December 31, 2019; patients aged ≥5 years; patients who were diagnosed with PTB; patients who lived in the study site for at least 6 months before diagnosis; patients who had available greenness data for residential addresses. Additionally, we excluded migrant patients who moved out of Zhengzhou during the study period and ethnic minority patients (Figure S1 in Multimedia Appendix 1).

### Assessment of Greenness and PM_2.5_

NDVI was used to estimate the level of greenness derived from the Moderate-Resolution Imaging Spectroradiometer [14]. Theoretically, the values of NDVI range from −1 to 1, with −1 to 0 representing bodies of water, 0 representing bare soil, and 0 to ＋1 representing healthy green vegetation; larger values represent levels of vegetative density [14]. The annual mean concentrations of ambient PM_2.5_ in Zhengzhou between January 1, 2012, and December 31, 2019, were estimated in the CHAP. The validation results were of high quality, with a cross-validation coefficient of determination (*R*^2^) of 0.92 for yearly predicted PM_2.5_ estimates; the corresponding root mean square errors of ground measurements were 10.76 μg/m^3^, and a mean absolute error was 6.32 µg m^−3^ on a daily basis [15]. The average NDVI and PM_2.5_ were calculated for each patient during the follow-up periods [10,13]. We assigned estimates of exposure to the greenness of 250 m and 500 m every 16 days and high-resolution (1 km) annual average PM_2.5_ data based on the patients’ current residential address information, which was geocoded into longitude and latitude [10].

### Outcome Measures

In this study, PTB retreatment was defined as the treatment given to patients who had irregular anti-TB treatment ≥1 month, experienced treatment failure, or had TB recurrence (a new clinical or microbiological PTB diagnosis in patients previously considered cured of PTB after their first episode) from January 1, 2012, to December 31, 2019 [17,18]. MDR-TB was defined as TB caused by bacteria that are at least resistant to rifampicin and isoniazid, the two major first-line anti-TB drugs [18,19]. According to the National Health Industry Standard on Diagnosis for Pulmonary Tuberculosis (WS 288‐2008 and WS 288‐2017) and the National Health Industry Standard on Classification for Pulmonary Tuberculosis (WS196-2001 and WS196-2017) in China, the diagnosis of PTB was based on the patient’s symptoms, chest x-rays, sputum smear microscopy, and culture results; the detailed description is in supplementary methods in Multimedia Appendix 2 [20,21]. The information of all patients with PTB was registered in a web-based reporting system at the Sixth Peoples Hospital of Zhengzhou (Zhengzhou Tuberculosis Prevention Institute), from diagnosis confirmation to retreatment during treatment follow-up from January 1, 2012, to December 31, 2019.

### Individual and Ground-Based Covariates

Potential individual covariates, including age, sex, occupation, drug resistance (MDR-TB), and county-level migrant patient, were adjusted. Besides, we controlled for potential ground-level traffic-related air pollution confounders as follows: distances to the nearest traffic roads (eg, national roads, highways, provincial roads, and living streets), road length and road density in the 500 m buffer around patient’s residential addresses, and the NTL index [13].

**Figure 1.**
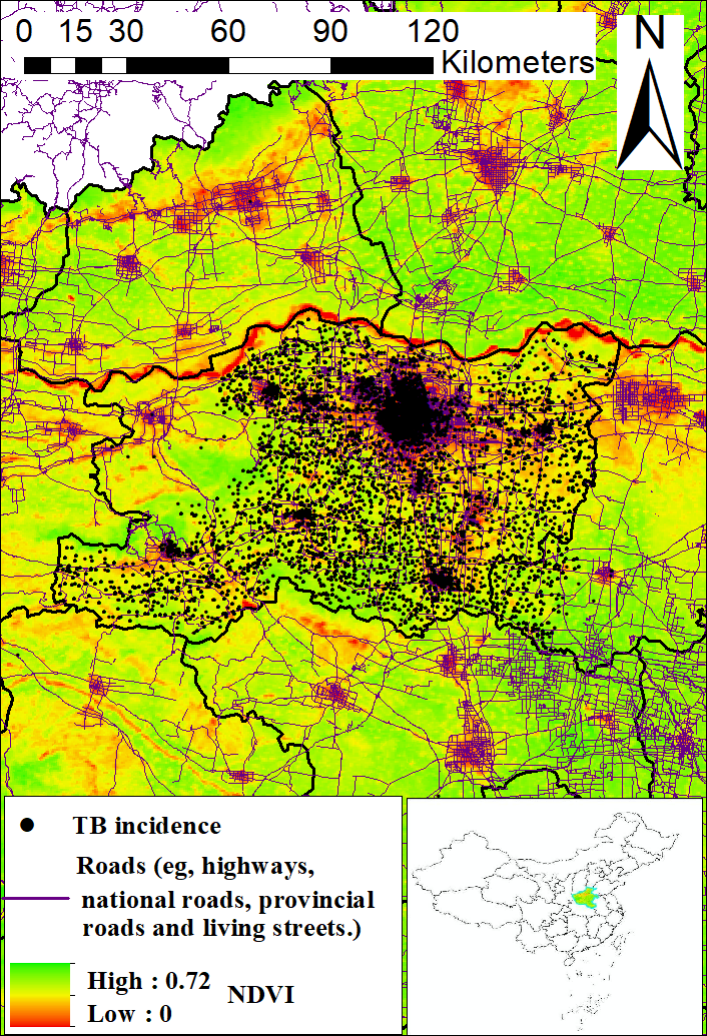
The distribution characteristics of tuberculosis (TB), traffic roads, and greenness (Normalized Difference Vegetation Index [NDVI]) in Zhengzhou, China.

Traffic data on national roads, highways, provincial roads, and living streets were obtained from OpenStreetMap road network data, which have been used in previous studies (Figure 1) [22]. Several studies show that NTL remote sensing is an important proxy for human socioeconomic space activities and energy consumption. Zhang et al [23] calculated the Prolonged Artificial Nighttime-light Dataset of China (PANDA) from 1984 to 2020 using an NTL convolutional long short-term memory network. The PANDA data, which have been used in previous studies, were publicly available from the National Tibetan Plateau Data Center. The spatial resolution of the PANDA dataset is approximately 1 km (30 arc s), with a value ranging from 0 to 63, which indicates a dimensionless quantity. Model assessments between the PANDA dataset and the original image showed that the root mean square error was 0.73, the coefficient of determination (*R*^*2*^) was 0.95, and the slope of pixels was 0.99, indicating that the quality of the dataset product was high [23].

### Statistical Analysis

Continuous variables with nonnormal distribution were expressed as median (IQRs), and normally distributed variables were expressed as mean (SDs). Categorical variables were expressed as numbers (percentages) for baseline characteristics. The associations of PM_2.5_ or greenness exposures with the risk of PTB retreatment were examined by hazard ratios (HRs) and 95% CIs, which were estimated by Cox proportional hazards regression models (HRs and 95% CIs were calculated per 10 μg/m^3^ and presented for annual mean PM_2.5_). We fitted multivariable Cox proportional hazards regression models with a priori–selected covariates.

The odds ratios (ORs) and 95% CIs were estimated by logistic regression models to examine the association of greenness exposures with the risk of drug resistance. We fitted 2 logistic regression models with a priori–selected covariates. The crude models (age-adjusted) and the multivariable models (fully adjusted) were fitted. A detailed description of adjusted covariates can be found in supplementary methods in Multimedia Appendix 2 .

PM_2.5_ or NDVI levels were stratified into 4 groups in all models according to the IQRs of PM_2.5_ or NDVI exposure levels, from low to high quintiles (Q1, Q2, Q3, and Q4), respectively. Sensitivity analyses were conducted by only including microbiologically confirmed PTB cases or TB recurrence. The dose-response associations of PM_2.5_ or greenness exposure levels with outcomes were assessed using a restricted cubic spline based on Cox proportional hazards regression models with 3 knots, and the nonlinearity was tested by Wald statistics [24]. All covariates in dose-response association analysis models were the same as the covariates in the previous multivariable Cox proportional hazards regression models. In addition, subgroup analyses were performed according to age, sex, occupation, drug resistance, annual average PM_2.5_ concentration, and the NDVI level. Statistical analyses were performed using ArcGIS 10.2 (Esri) and R software (version 4.0.5; R Project for Statistical Computing) with the analysis packages survival (version 3.2‐10), data.table (version 1.14.2), rms (version 6.2‐0), ggplot2 (version 3.3.5), raster (version 3.5‐15), rasterVis (version 0.51.2) and car (version 3.0‐12). All statistical tests were 2-sided, and a *P* value <.05 was considered statistically significant.

### Ethics Approval

This study was approved by the Henan Provincial Infectious Disease Hospital Ethical Review Board (IEC-KYM-2024‐07) and was exempt from obtaining informed consent, as it involved secondary analysis of registeration data [25].

## Results

### Participant Characteristics

A total of 26,482 patients with a mean age of 46.86 (SD 19.52) years registered between 2012 and 2019 in Zhengzhou were included in the analysis. Among the study participants, there were 1542 PTB retreatment (1372 recurrence PTB) cases during the follow-up period, with a median follow-up time of 1523 days per patient. Characteristics of the participants in the PTB retreatment analysis are presented in Table 1 and Figure 1.

**Table 1.** Characteristics of the study population.

Characteristics		Values (N=26,482)
**Age at diagnosis date (years), n (%)**		
	5‐39.9	10,352 (39.09)
	40‐59.9	8181 (30.89)
	≥60	7949 (30.02)
	Missing data	NA^a^
**Sex, n (%)**		
	Female	7918 (29.90)
	Male	18,564 (70.10)
	Missing data	NA
**Occupation, n (%)**		
	Government, education, and retired	3243 (12.25)
	Agriculture	15,561 (58.76)
	Industry	1209 (4.57)
	Others	6469 (24.43)
	Missing data	NA
**Drug resistant, n (%)**		
	Yes	420 (1.58)
	No	26,062 (98.41)
	Missing data	NA
**TB^b^ treatment classification, n (%)**		
	Primary	24,940 (94.18)
	Retreatment	1542 (5.82)
	Missing data	NA
**NDVI** ^**c**^		
	Median (IQR)^d^	0.33 (0.21‐0.42)
	Missing data	NA
**PM_2.5_ (μg/m^3^)^e^**		
	Median (IQR)	79.54 (77.63‐81.40)
	Missing data	NA
**Nighttime light**		
	Median (IQR)	29 (9‐54)
	Missing data	NA
**Distance to the nearest roads (km)**		
	Median (IQR)	0.17 (0.06‐0.51)
	Missing data	NA
**Road length (km)**		
	Median (IQR)	3.29 (1.63‐7.10)
	Missing data, n (%)	7448 (28.12)
**Road density (counts per km** ^**2**^ **)**		
	Median (IQR)	4.28 (2.12‐9.22)
	Missing data, n (%)	7448 (28.12)

a NA: not available.

b TB: tuberculosis.

c NDVI: Normalized Difference Vegetation Index.

d NDVI within 500 m buffers.

e PM_2.5_: fine particulate matter with an aerodynamic diameter of 2.5 μm or less.

### Greenness and PM_2.5_ Exposures

The mean annual PM_2.5_ was 79.13 (SD 2.71), ranging from 68.58 to 85.35 μg/m^3^ between 2012 and 2019 (Table 2). The medians of NDVI within 250 m and 500 m buffers were 0.33 (IQR 0.21‐0.42) and 0.30 (IQR 0.20‐0.39), respectively (Table 3). There was a moderate negative correlation between NDVI and the annual average of PM_2.5_ (*r*=−0.66; *P*<.001) and a strong negative correlation between NDVI and NTL (*r*=−0.91; *P*<.001). The median of NTL was 29.00 (IQR 9-54). In this study, 113,228 patients were exposed to lower NTL. There was a strong positive correlation between NTL and the annual average of PM_2.5_ (*r*=0.70; *P*<.001).

**Table 2.** The association between the annual mean level of fine particulate matter with an aerodynamic diameter of 2.5 μm or less (PM_2.5_) and the retreatment of tuberculosis for the level of greenness around the patient’s residence (adjusted hazard ratios [HRs] per 10 µg/m^3^ increase in annual mean PM_2.5_) in 4 quintile (Q) groups. *P* for trend was .003 for PM_2.5_ model 1 and 0.01 for PM_2.5_ model 2; *P* for trend was <.001 and .07 for the Normalized Difference Vegetation Index (NDVI) model 1 and model 2, respectively.

Exposure (PM _2.5_)		Mean (SD)	Model 1^a^		Model 2^b^	
			Age-adjusted HR (95% CI)	*P* value	Fully adjusted HR (95% CI)	*P* value
All participants (per 10 µg/m^3^)		79.13 (2.71)	2.16 (1.79‐2.59)	<.001	1.97 (1.34‐2.83)	<.001
**PM_2.5_^c^**						
	Q1	75.27 (1.79)	1 (reference)	—^d^	1 (reference)	—
	Q2	78.58 (0.57)	2.00 (1.58‐2.52)	<.001	2.50 (1.77‐3.52)	<.001
	Q3	80.57 (0.62)	2.64 (2.12‐3.27)	<.001	2.63 (1.91‐3.60)	<.001
	Q4	81.96 (0.55)	2.12 (1.74‐2.58)	<.001	1.80 (1.31‐2.48)	<.001
**NDVI^e^ (500 -mm buffer)^f^**						
	Q1	81.53 (0.81)	7.30 (2.16‐25.04)	.002	1.62 (0.31‐7.30)	.58
	Q2	79.94 (2.22)	1.97 (1.22‐3.39)	.006	1.97 (1.00‐3.71)	.05
	Q3	78.00 (2.53)	1.63 (1.00‐2.84)	.05	1.34 (0.60‐2.83)	.54
	Q4	77.04 (2.33)	2.16 (1.34‐3.39)	<.001	1.79 (1.00‐3.70)	.05

a Cox model was adjusted for age at the diagnosis date.

b Cox model was adjusted for age at the diagnosis date, sex, occupation, county-level migrant population, drug resistance, NDVI, nighttime light, distance to the nearest roads, road length, and road density.

c According to the interquartile range of PM_2.5_ exposure levels from low to high, participants were divided into Q1, Q2, Q3, and Q4 groups.

d Not applicable.

e NDVI: N=26,482.

f According to the interquartile range of NDVI exposure levels from low to high, participants were divided into Q1, Q2, Q3, and Q4 groups, and the NDVI was not adjusted for this model.

**Table 3.** The association between the level of greenness and the retreatment of tuberculosis in 4 quintile (Q) groups. *P* for trend was .37 and .16 for the Normalized Difference Vegetation Index (NDVI; 250 m buffer) in model 1 and 2; *P* for trend was .86 and .58 for NDVI (500 m buffer) in model 1 and 2.

Exposure (NDVI^a^)		Median (IQR)	Model 1^b^		Model 2^c^	
			Age-adjusted HR^d^ (95% CI)	*P* value	Fully adjusted HR (95% CI)	*P* value
**NDVI (250 m buffer)**						
	All participants	0.33 (0.21‐0.42)	—^e^	—	—	—
	Q1	0.17 (0.16‐0.19)	1 (reference)	—	1 (reference)	—
	Q2	0.25 (0.22‐0.27)	0.71 (0.62‐0.82)	<.001	0.76 (0.64‐0.89)	.001
	Q3	0.34 (0.32‐0.37)	0.53 (0.45‐0.61)	<.001	0.75 (0.58‐0.98)	.03
	Q4	0.44 (0.41‐0.47)	0.79 (0.69‐0.90)	<.001	0.91 (0.66‐1.25)	.55
**NDVI (500 m buffer)**						
	All participants	0.30 (0.20‐0.39)	—	—	—	—
	Q1	0.18 (0.17‐0.19)	1 (reference)	—	1 (reference)	—
	Q2	0.26 (0.24‐0.30)	0.70 (0.61‐0.81)	<.001	0.75 (0.63‐0.89)	.002
	Q3	0.38 (0.36‐0.40)	0.65 (0.57‐0.75)	<.001	0.55 (0.40‐0.77)	<.001
	Q4	0.45 (0.41‐0.48)	0.73 (0.64‐0.84)	<.001	0.98 (0.67‐1.42)	.93

a NDVI: N=26,482.

b Cox model was adjusted for age at the diagnosis date.

c Cox model was adjusted for age at the diagnosis date, sex, occupation, county-level migrant population, drug resistance, annual average fine particulate matter with an aerodynamic diameter of 2.5 μm or less (PM_2.5_) concentration, nighttime light, distance to the nearest roads, road length, and road density.

d HR: hazard ratio.

e Not applicable.

### PM_2.5_ Exposures and PTB Retreatment

As shown in Table 2, exposure to PM_2.5_ was significantly associated with the increased risk of PTB retreatment in both age-adjusted (HR 2.16, 95% CI 1.79‐2.59 per 10 μg/m^3^ increase in PM_2.5_) and fully adjusted models (HR 1.97, 95% CI 1.34‐2.83 per 10 μg/m^3^ increase in PM_2.5_) for the full study patients. NDVI and NTL exhibited a modifying effect on the association between PM_2.5_ exposure and the risk of PTB retreatment. (Table 2 and Table S1 in Multimedia Appendix 2). We did not observe significant associations between exposure to PM_2.5_ and the risk of PTB retreatment in patients living in higher NDVI and higher NTL areas (Table 2 and Table S1 in Multimedia Appendix 2).

### Greenness Exposures and PTB Retreatment

Table 3 presents HRs (95% CIs) for PTB retreatment in age-adjusted models and fully adjusted models. In the multivariate analyses, compared with group Q1 (reference group) with the lowest quintile of greenness, patients exposed to the second (Q2) and third quintile (Q3) of greenness within the 500 m buffer had lower odds of PTB retreatment (HR 0.75, 95% CI 0.63‐0.89 and HR 0.55, 95% CI 0.40‐0.77, respectively); details of the fully adjusted HR (95% CI) for the 250 m and 500 m buffers are listed in Table 3. Similar associations were observed in sensitivity analysis after the inclusion of patients with microbiologically confirmed PTB results or TB recurrence (Table S2-S3 in the Multimedia Appendix 2). No statistically significant association between the highest quintile of greenness and PTB retreatment was observed (Table 3).

In addition, we examined the effect modification by age (5‐39.9 years, 40‐59.9 years, and ≥60 years), sex (male and female), occupation (agriculture, industry, government, education, retired, and others), drug resistance (yes and no), and annual average PM_2.5_ concentration (low: <79.54 μg/m^3^ and high: ≥79.54 μg/m^3^) among patients; living in areas with higher greenness is more likely to benefit younger patients (5‐39.9 years); female patients; those working in the government, education sector, or retired; those working indoors; drug-sensitive patients; and those having lower PM_2.5_ exposure (Tables S4-S8 in Multimedia Appendix 2). In the effect modifications analyses of NTL areas, compared to the first quintile (Q1) of greenness within the 500 m buffer, a negative association was observed in reducing the risk of PTB retreatment for patients living in higher NTL areas (Q3: HR 0.70, 95% CI 0.57‐0.87; Q4: HR 0.73, 95% CI,0.57‐0.94); meanwhile, a positive association was observed for patients living in lower NTL areas (Q3: HR 1.69, 95% CI 1.26‐2.27; Q4: HR 1.78, 95% CI 1.30‐2.43; Table 4). Lastly, we did not observe significant associations between higher greenness exposure and the risk of PTB drug-resistant in the patients, including those with PTB retreatment (Table S9 in Multimedia Appendix 2).

**Table 4.** The association between the level of greenness with 500 m buffers around residential addresses and the retreatment of tuberculosis for nighttime light (NTL) in 4 quintile (Q) groups. *P* for trend was <.001 for both model 1 and 2 in low NTL; *P* for trend was <.001 and .002 for model 1 and 2 in high NTL, respectively.

Exposure (NDVI^a^, 500 m buffer）		Model 1^b^		Model 2^c^	
		Age-adjusted HR^d^ (95% CI)	*P* value	Fully adjusted HR (95% CI)	*P* value
**Low NTL^e^** ** (n=13,228)**					
	Q1	1 (reference)	—	1 (reference)	—
	Q2	1.01 (0.81‐1.27)	.95	1.00 (0.72‐1.39)	.98
	Q3	1.36 (1.10‐1.67)	.006	1.69 (1.26‐2.27)	<.001
	Q4	1.48 (1.20‐1.82)	<.001	1.78 (1.33‐2.49)	<.001
**High NTL^e^ (n=13,254)**					
	Q1	1 (reference)	—^d^	1 (reference)	—
	Q2	0.80 (0.67‐0.95)	.01	0.84 (0.70‐1.00)	.05
	Q3	0.63 (0.52‐0.76)	<.001	0.70 (0.57‐0.87)	.001
	Q4	0.60 (0.50‐0.73)	<.001	0.73 (0.58‐0.95)	.02

a NDVI: Normalized Difference Vegetation Index (N=26,482).

b Cox model was adjusted for age at the diagnosis date.

c Cox model was adjusted for age at the diagnosis date, sex, occupation, county-level migrant population, drug resistance, annual average fine particulate matter with an aerodynamic diameter of 2.5 μm or less (PM_2.5_) concentration, distance to the nearest roads, road length, and road density.

d HR: hazard ratio.

e Ground-level NTL: yearly average NTL index as a proxy for socioeconomic level and urbanization, using the median value of 29 as the low-high cutoff value.

### Dose-Response Associations

**Figure 2.**
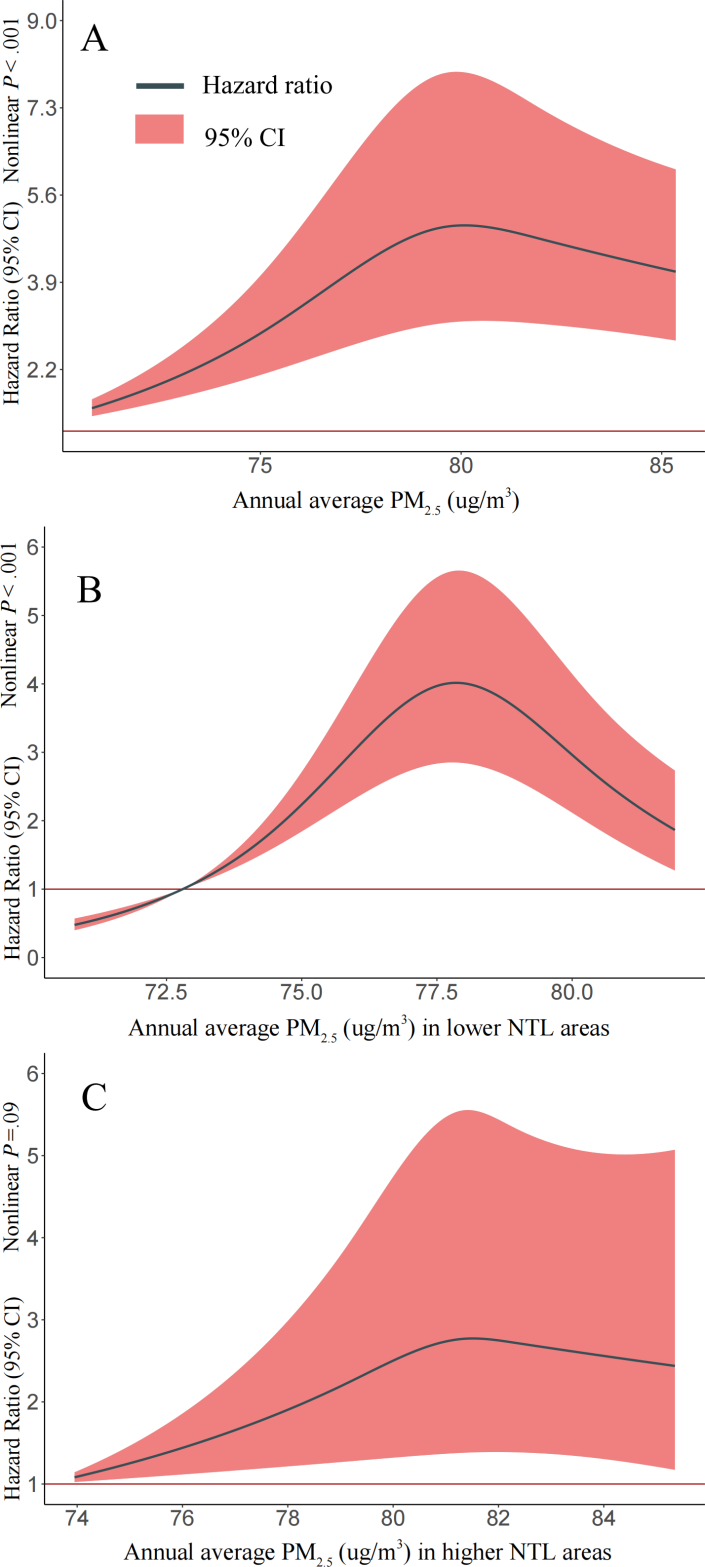
Dose-response associations between annual mean fine particulate matter with an aerodynamic diameter of 2.5 μm or less (PM_2.5_) and the risk of tuberculosis retreatment (2012‐2019). The multivariable-adjusted hazard ratios are shown for the associations between PM_2.5_ levels and the risk of tuberculosis retreatment in different nighttime light (NTL) areas (parts A, B, and C). The Cox model was adjusted for age at the diagnosis date, sex, occupation, county-level migrant population, drug resistance, Normalized Difference Vegetation Index, NTL, distance to the nearest roads, road length, and road density. Green curves and red areas show predicted hazard ratios and 95% CIs, respectively.

There were nonlinearity associations between annual average PM_2.5_ exposure and PTB retreatment among patients for the full retrospective study (Figure 2A; nonlinear *P*<.001) and patients living in lower NTL areas (Figure 2B; nonlinear *P*<.001); meanwhile, a linear association between annual average PM_2.5_ exposure and PTB retreatment among patients living in higher NTL areas was observed (Figure 2C; nonlinear *P*=.09).

**Figure 3.**
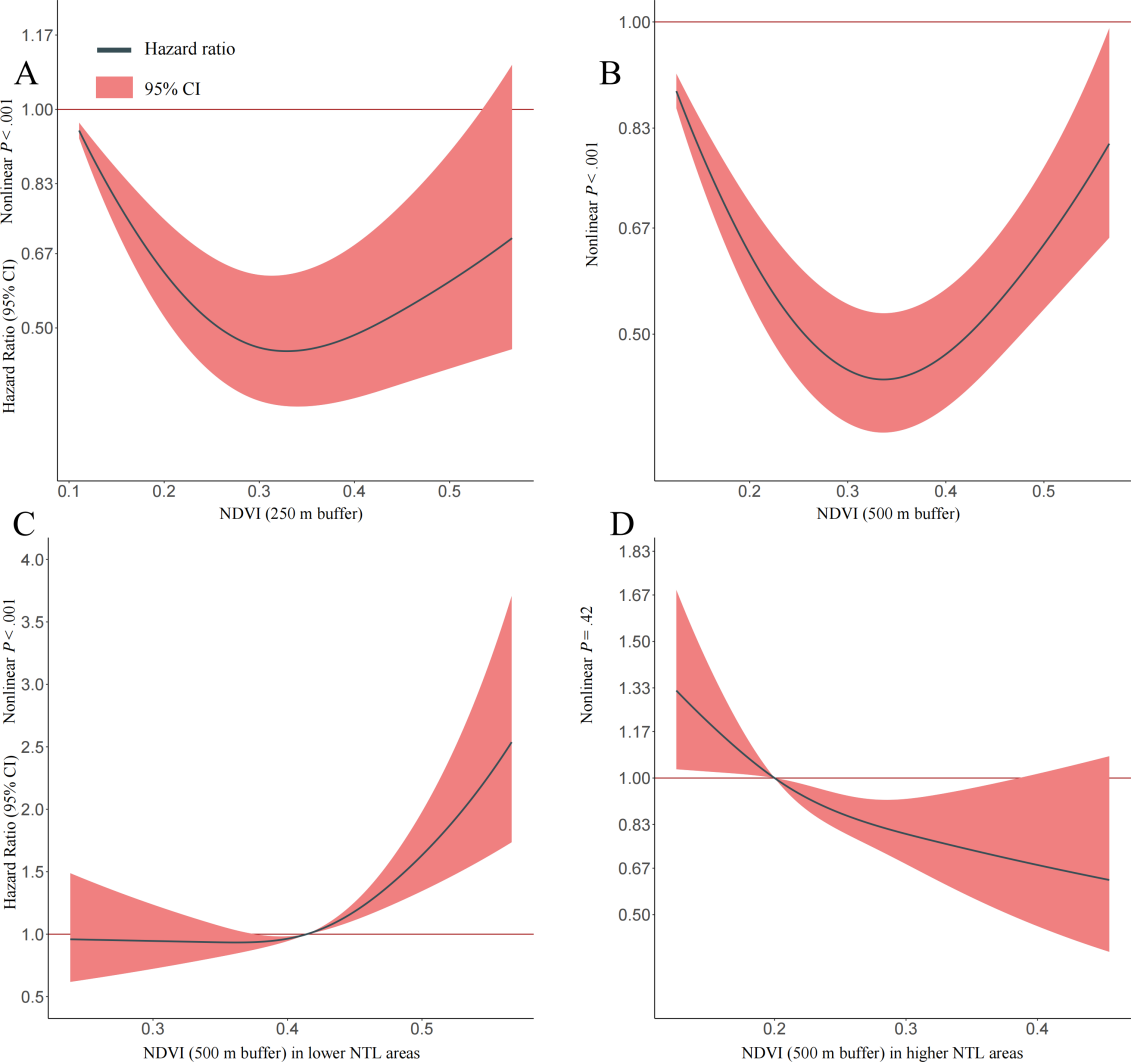
Dose-response association between greenness exposure and the risk of tuberculosis retreatment (2012‐2019). The multivariable-adjusted hazard ratios are shown for the associations between greenness exposure levels and the risk of tuberculosis retreatment (parts A and B) in different nighttime light (NTL) areas (parts C and D). The Cox model was adjusted for age at the diagnosis date, sex, occupation, county-level migrant population, drug resistance, annual average fine particulate matter with an aerodynamic diameter of 2.5 μm or less (PM_2.5_) concentration, NTL, distance to the nearest roads, road length, and road density. Green curves and red areas show predicted hazard ratios and 95% CIs, respectively. NDVI: Normalized Difference Vegetation Index.

The dose-response curves shown in Figure 3 suggested nonlinearity associations between greenness exposure and PTB retreatment among patients for the full retrospective study (Figure 3A and 3B; nonlinear *P*<.001) within the 250 m and 500 m buffer around the patient’s address and patients living in lower NTL areas (Figure 3C; nonlinear *P*<.001). In addition, the linear association between greenness exposure and PTB retreatment among patients living in higher NTL areas was observed (Figure 3D; nonlinear *P*=.42).

## Discussion

### Principal Findings

To our knowledge, this is the first study investigating the associations of PM_2.5_ and greenness with the retreatment of PTB in a population-based retrospective study and well control for potential covariates, including individual and ground-based factors associated with the risk of PTB retreatment.

The findings suggest that PM_2.5_ exposure was significantly associated with the increased risk of PTB retreatment and the exposure to higher greenness was associated with decreased risk of PTB retreatment. Moreover, this study adds evidence that greenness exposure attenuated the association between PM_2.5_ exposure and PTB retreatment. Our findings enhance our knowledge underpinning control of ambient air pollution, improving greenness, and PTB management.

Most previous research focused on the effect of newly diagnosed active TB, and there are limited data on the association of PM_2.5_ exposure with PTB retreatment [4,5]. A systematic literature review reported a positive association between PM_2.5_ exposure and the risk of TB outcomes, including TB incidence, hospital admissions, and death [4]. Results from a recent nationwide modeling study conducted by Zhu et al [12] indicated that long-term PM_2.5_ exposure was positively associated with PTB incidence in China. Besides, consistent with a recent study conducted by Liu et al [26], which has suggested that both short- and long-term exposures to outdoor PM_2.5_ were significantly associated with the risk of TB recurrence in Shandong, China, our study identified a significant positive association between PM_2.5_ exposure and PTB retreatment. The possible biological mechanisms underlying the adverse effects of ambient PM_2.5_ exposure against PTB are as follows: (1) air pollution, especially ambient fine particulate matter, could reduce lung defense functions, which may lead to the development of pulmonary diseases [27]; (2) PM_2.5_ exposure could contribute to the inflammation of cytotoxicity of T cells in a macrophage-dependent manner and decrease the expression of interferon-gamma, which might result in the progression of PTB [28,29]; (3) inflammation caused by PM_2.5_ and oxidative stress in epithelial cells and macrophages may reduce the immune response, which increases the susceptibility to TB [30,31]; (4) the accumulation of iron through PM_2.5_ consisting of transition metals may contribute to iron availability and provide a good microenvironment for the proliferation of MTB [32,33].

Additionally, the above mechanisms might be more prominent in patients with a history of TB [34]. Therefore, long-term exposure to ambient PM_2.5_ may accelerate the progression of TB retreatment through the above-mentioned mechanisms and associations. Moreover, we observed that greenness attenuated the association between PM_2.5_ exposure and the risk of PTB retreatment, which is consistent with a previous study [12]. There was a moderate negative correlation between NDVI and the annual average of PM_2.5_ (*r*=−0.7; *P*<.001); this correlation trend was consistent with a previous study assessing the effect modification of greenness on PM_2.5_-associated all-cause mortality among patients with MDR-TB and may account for the effect modification of greenness on PM_2.5_ and PTB retreatment, but we did not find a previous study that reported the correlation between NTL and PM_2.5_ [35]. Therefore, further research on the precise mechanisms underlying associations between PM_2.5_ and PTB retreatment is required.

Most previous studies focused on urban residential greenness and various health outcomes, including mental health [6], weight management [7], sleep duration [8], cardiovascular health [9], and mortality [10]. There is no research assessing the association between urban residential greenness and PTB retreatment so far. We observed that exposure to the relatively high greenness had lower odds of PTB retreatment, but no statistically significant association was identified between the highest quintile of greenness and PTB retreatment. Interestingly, our further analysis of stratified patients by NTL areas found a negative association in reducing the risk of PTB retreatment for patients living in higher NTL areas but a positive association for patients living in lower NTL areas.

Consistent with a recent study in China [13], a strong negative correlation between NDVI and NTL was observed in our study. NTL is an important proxy for socioeconomic status, gross domestic product, and urbanization development [36,37]. Higher greenness exposure in China has been associated with a relatively lower gross domestic product, socioeconomic status, and medical conditions, including PTB treatment and management, due to urbanization development, which may explain the attenuated association between the highest quintile of greenness and the risk of PTB retreatment and the effect modification of NTL on greenness associated PTB retreatment [13,36,37]. Significant associations between higher greenness exposure and the risk of MDR-TB in all patients and in patients with PTB retreatment were not observed in our study. Multiple factors may contribute to drug resistance, including low BMI, lower economic status, and smoking, which unfortunately are unavailable in our data. Future detailed and effective investigations, such as prospective multiple centers cohorts with large sample sizes and multiple risk factors, are needed to further validate our results. Actively exploring potential factors associated with MDR-TB may contribute to the prevention and management of MDR-TB [38].

Therefore, elucidating potential environmental predictors or factors associated with the retreatment of PTB may help PTB control by exploring more appropriate public health measures to reduce the incidence of PTB, such as controlling high levels of air pollution and improving the environmental amount of greenness. However, further studies with detailed information on economic development, PTB treatment, PTB management, and environmental factors to assess the associations between air pollution, greenness, and PTB retreatment are needed. The strengths of our study included the relatively detailed data on demographic characteristics and environment, which allowed us to conduct fully adjusted models and stratified analyses. In addition, we stratified our analyses by NTL, which represents levels of economic activities and urbanization to reduce the potential self-selection bias in our analysis.

### Limitations

Several limitations should be kept in mind when interpreting our results. First, the NDVI data describe only the amount and presence of vegetation and cannot represent information on the specific type or quality of vegetation, which does not allow for distinctions between urban green plants or rural agricultural areas. Second, although we excluded all migrant patients in our analyses, residential self-selection bias, which could be affected by socioeconomic status, is another issue. We even stratified our analyses by NTL, representing levels of economic activities and urbanization, to control for such potential bias. Third, although we adjusted several potential confounding factors in our multivariable model, confounding bias could not be completely excluded. Fourth, TB registration data were significantly influenced and thus lacked representativeness during the outbreak of COVID-19. Therefore, we did not include the data between 2020 and 2023 in our study.

### Conclusions

Consistently, PM_2.5_ exposure was observed to be significantly associated with the increased risk of PTB retreatment in our study population. What is more valuable, this study is the first population-based study to report that higher greenness was associated with a decreased risk of PTB retreatment, but with an increased risk of PTB retreatment for patients living in lower NTL areas. With the development of urbanization in China, our findings provide evidence for city planners and health policy makers that controlling ambient air pollution and improving residential greenness may contribute to the reduction of PTB retreatment.

## Supplementary material

10.2196/50244 Multimedia Appendix 1 Flowchart of the study population.

10.2196/50244 Multimedia Appendix 2 Additional statistics (supplementary methods, subgroup analysis, and sensitivity analysis).

## Abbreviations

CHAP: China High Air Pollutants
HR: hazard ratio
MDR-TB: multidrug-resistant tuberculosis
MTB: *Mycobacterium tuberculosis*
NDVI: Normalized Difference Vegetation Index
NTL: nighttime light
OR: odds ratio
PANDA: Prolonged Artificial Nighttime-light Dataset of China
PM_2.5_: fine particulate matter with an aerodynamic diameter of 2.5 μm or less
PTB: pulmonary tuberculosis
SPPTB: smear-positive pulmonary tuberculosis
TB: tuberculosis
