# Supplementary figures and images for: Association of Fine Particulate Matter and Residential Greenness With Risk of Pulmonary Tuberculosis Retreatment: Population-Based Retrospective Study
Source: JMIR Public Health Surveill. 2024 Aug 12;10:e50244. doi: 10.2196/50244 (PMC11337066; doi:10.2196/50244)

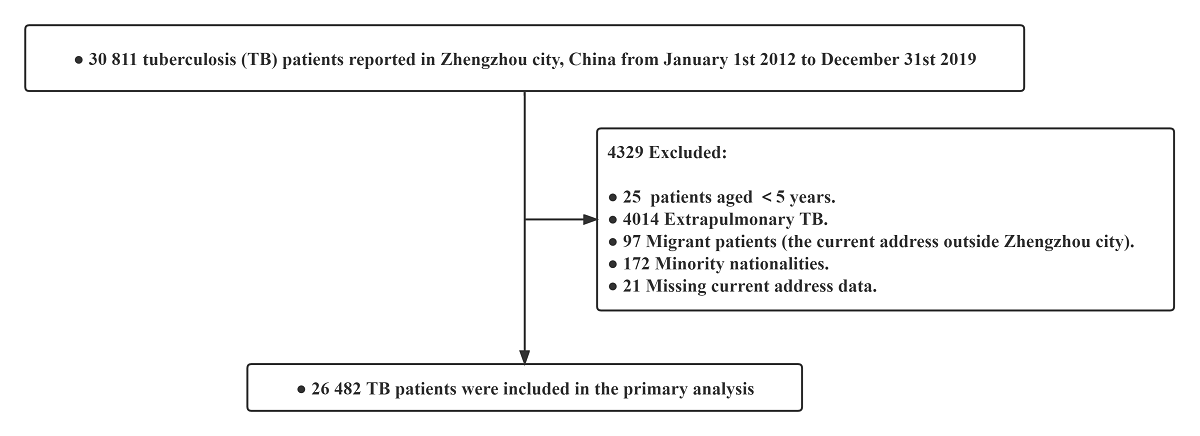

Supplement: Multimedia Appendix 1 [file publichealth-v10-e50244-s001.png]
