# Supplementary material for: Association of Fine Particulate Matter and Residential Greenness With Risk of Pulmonary Tuberculosis Retreatment: Population-Based Retrospective Study
Source: JMIR Public Health Surveill. 2024 Aug 12;10:e50244. doi: 10.2196/50244 (PMC11337066; doi:10.2196/50244)
Supplement: Multimedia Appendix 2 [file publichealth-v10-e50244-s002.docx]

**Association of Fine Particulate Matter and Residential Greenness with Risk of Retreatment of Pulmonary Tuberculosis: A Population-based Retrospective Study**

**Supplementary materials. (Supplementary methods; Table S1-8)**

**Supplementary methods**

The crude model (age-adjusted) only adjusted for a patient’s age at the diagnosis date. The multivariable model (fully adjusted) was further adjusted for sex, occupation, county-level migrant population, drug resistance, annual average PM_2.5_ concentration or the Normalized Difference Vegetation Index (NDVI), the nighttime light (NTL) index, distance to the nearest roads, road length and road density.

The odds ratios (ORs) and 95% CIs were estimated by logistic regression models to examine the association of greenness exposures with the risk of drug resistance. We fitted 2 logistic regression models with a priori-selected covariates. The crude model (age-adjusted) adjusted for a patient’s age at the diagnosis date. In the multivariable model (fully adjusted), we additionally adjusted for sex, occupation, county-level migrant population, annual average PM_2.5_ concentration, NDVI, NTL, distance to the nearest roads, road length and road density.

PM_2.5_/NDVI levels were stratified into 4 groups in all the models according to the IQRs of PM_2.5_/NDVI exposure levels from low to high (Q1, Q2, Q3 and Q4), respectively. Sensitivity analyses were conducted by only including microbiologically confirmed PTB patients. The dose-response associations of PM_2.5_ or greenness exposure levels with outcomes were assessed using a restricted cubic spline (RCS) based on Cox proportional hazards regression models with three knots at the 10th, 50th and 90th percentiles of PM_2.5_ or greenness exposure levels, and the nonlinearity was tested by Wald statistics. For the follow-up time of initial PTB treatment patients, the calculation of time difference is between the registration time of the first treatment and December 31, 2019, and for PTB retreatment patients, the calculation of time difference is between January 1, 2012 or the registration time of the first treatment, and the registration time of retreatment.

According to two national editions of standards for the diagnosis of tuberculosis (TB) (WS 288-2008 and WS 288-2017) in China, the diagnosis of pulmonary tuberculosis (PTB) was mainly based on the patient’s symptoms, chest X-rays, sputum smear microscopy, and culture results. multidrug-resistant tuberculosis (MDR-TB) was defined as TB caused by bacteria that are at least resistant to rifampicin and isoniazid, the two major first-line anti-TB drugs. The drug-susceptibility testing mainly includes molecular drug resistance testing and Mycobacterium tuberculosis culture. The detailed diagnosed description of PTB and MDR-TB were as follows in four national editions of standards for the diagnosis of PTB (WS 288-2008 and WS 288-2017) and classification of PTB (WS196-2001 and WS196-2017) in China:

1) National Health and Family Planning Commission of the People’s Republic of China. Diagnostic criteria for pulmonary tuberculosis (WS 288-2008). 2008.

2) National Health and Family Planning Commission of the People’s Republic of China. Diagnosis for pulmonary tuberculosis (WS 288-2017). 2017.

3) National Health and Family Planning Commission of the People’s Republic of China. Classification standards of tuberculosis (WS196-2001). 2001.

4) National Health and Family Planning Commission of the People’s Republic of China. Classification standards of tuberculosis (WS196-2017). 2017.

**Table S1** **Adjusted hazard ratios (HRs) per 10 µg/m^3^ increase in annual mean PM_2.5_ associated with the retreatment of tuberculosis according to nighttime light.**

|  |  | **Mean (SD)** |  | **Model^a^** |  | **Model^b^** |
| --- | --- | --- | --- | --- | --- | --- |
| **Exposure （PM_2.5_）** |  |  |  | **Age-adjusted HR (95% CI)** |  | **Fully adjusted HR (95% CI)** |
| All participants |  | 79.13 (2.71) |  | 2.16 (1.79-2.59) |  | 1.97 (1.34-2.83) |
| **Low NTL^c^, n = 13 228** |  |  |  |  |  |  |
| All participants^d^ |  | 77.39 (2.49) |  | 1.79 (1.34-2.59) |  | 1.79 (1.10-2.83) |
| Q1 |  | 73.92 (1.42) |  | 1 [Reference] |  | 1 [Reference] |
| Q2 |  | 77.02 (0.51) |  | 1.91 (1.50-2.42) |  | 2.05 (1.43-2.96) |
| Q3 |  | 78.30 (0.37) |  | 2.66 (2.12-3.35) |  | 2.69 (1.90-3.81) |
| Q4 |  | 80.30 (0.91) |  | 1.33 (1.03-1.72) |  | 1.35 (0.91-2.02) |
| **High NTL^c^, n = 13 254** |  |  |  |  |  |  |
| All participants^d^ |  | 80.86 (1.58) |  | 2.16 (2.15-6.19) |  | 1.79 (0.90-3.39) |
| Q1 |  | 78.72 (1.50) |  | 1 [Reference] |  | 1 [Reference] |
| Q2 |  | 80.82 (0.38) |  | 1.30 (1.05-1.62) |  | 1.10 (0.86-1.41) |
| Q3 |  | 81.55 (0.15) |  | 1.79 (1.47-2.20) |  | 1.29 (1.00-1.67) |
| Q4 |  | 82.32 (0.57) |  | 1.54 (1.25-1.90) |  | 1.15 (0.89-1.48) |

^a^Cox model was adjusted for age at the diagnosis date.

^b^Cox model was adjusted for age at the diagnosis date, sex, occupation, county-level migrant population, drug resistance, annual average PM_2.5_ concentration, distance to the nearest roads, road length and road density.

^c^Ground-level NTL: yearly average nighttime light index as a proxy for socio-economic level and urbanization, using the median value of 29 as the low-high cutoff.

^d^According to the interquartile range of PM_2.5_ exposure levels from low to high, the participants were divided into Q1, Q2, Q3 and Q4 groups. (N=26 482)

**Table S2** **Adjusted hazard ratios (HRs) of the retreatment of tuberculosis according to greenness exposure among microbially confirmed cases. (N=8205)**

|  |  | **No. of** |  | **TB** **retreatment**  **n (%)** |  |  | **Model^a^** |
| --- | --- | --- | --- | --- | --- | --- | --- |
| **Exposure （NDVI）** |  | **participants** |  |  |  |  | **Fully adjusted HR (95% CI)** |
| **NDVI (500-m buffer)** |  |  |  |  |  |  |  |
| Q1 |  | 2051 |  | 186 (9.07) |  |  | 1 [Reference] |
| Q2 |  | 2050 |  | 157 (7.66) |  |  | 0.78 (0.62-0.98) |
| Q3 |  | 2055 |  | 169 (8.22) |  |  | 0.84 (0.56-1.27) |
| Q4 |  | 2049 |  | 203 (9.91) |  |  | 1.33 (0.78-2.27) |
| *P* for Trend |  |  |  |  |  |  | 0.307 |

According to the interquartile range of normalized difference vegetation index (NDVI) exposure levels from low to high, the participants were divided into Q1, Q2, Q3 and Q4 groups.

^a^ Cox model was adjusted for age at the diagnosis date, sex, occupation, county-level migrant population, drug resistance, annual average PM_2.5_ concentration, nighttime light, distance to the nearest roads, road length and road density.

**Table S3 Adjusted hazard ratios (HRs) of the recurrence of tuberculosis according to greenness exposure. (N=26 312)**

|  |  | **No. of** |  | **TB** **retreatment**  **n (%)** |  |  | **Model^a^** |
| --- | --- | --- | --- | --- | --- | --- | --- |
| **Exposure （NDVI）** |  | **participants** |  |  |  |  | **Fully adjusted HR (95% CI)** |
| **NDVI (500-m buffer)** |  |  |  |  |  |  |  |
| Q1 |  | 6595 |  | 474 (7.19) |  |  | 1 [Reference] |
| Q2 |  | 6597 |  | 305 (4.62) |  |  | 0.75 (0.63-0.89) |
| Q3 |  | 6545 |  | 211 (3.22) |  |  | 0.60 (0.43-0.85) |
| Q4 |  | 6575 |  | 382 (5.81) |  |  | 1.27 (0.86-1.89) |
| *P* for Trend |  |  |  |  |  |  | 0.761 |

According to the interquartile range of normalized difference vegetation index (NDVI) exposure levels from low to high, the participants were divided into Q1, Q2, Q3 and Q4 groups.

^a^ Cox model was adjusted for age at the diagnosis date, sex, occupation, county-level migrant population, drug resistance, annual average PM_2.5_ concentration, nighttime light, distance to the nearest roads, road length and road density.

**Table S4** **Adjusted hazard ratios (HRs) of the retreatment of tuberculosis with greenness exposure (500 m buffers around residential addresses) according to age.**

|  |  | **Model^a^** |
| --- | --- | --- |
| **Exposure （NDVI, 500-m buffer）** |  | **Fully adjusted HR (95% CI)** |
| **Age 5-39**.**9 (years), n = 10 352** |  |  |
| Q1 |  | 1 [Reference] |
| Q2 |  | 0.75 (0.54-1.05) |
| Q3 |  | 0.45 (0.25-0.83) |
| Q4 |  | 0.53 (0.24-0.75) |
| *P* for Trend |  | 0.996 |
| **Age 40-59**.**9 (years), n = 8181** |  |  |
| Q1 |  | 1 [Reference] |
| Q2 |  | 0.78 (0.59-1.02) |
| Q3 |  | 0.54 (0.30-0.96) |
| Q4 |  | 0.73 (0.37-1.41) |
| *P* for Trend |  | 0.267 |
| **Age ≥60 (years), n = 7949** |  |  |
| Q1 |  | 1 [Reference] |
| Q2 |  | 0.76 (0.57-1.02) |
| Q3 |  | 0.50 (0.30-0.84) |
| Q4 |  | 1.08 (0.62-1.90) |
| *P* for Trend |  | 0.105 |

According to the interquartile range of normalized difference vegetation index (NDVI) exposure levels from low to high, the participants were divided into Q1, Q2, Q3 and Q4 groups. (N=26 482)

^a^Cox model was adjusted for sex, occupation, county-level migrant population, drug resistance, annual average PM_2.5_ concentration, nighttime light, distance to the nearest roads, road length and road density.

**Table S5** **Adjusted hazard ratios (HRs) of the retreatment of tuberculosis with greenness exposure (500 m buffers around residential addresses) according to sex.**

|  |  | **Model^a^** |
| --- | --- | --- |
| **Exposure （NDVI, 500-m buffer）** |  | **Fully adjusted HR (95% CI)** |
| **Male, n = 18 564** |  |  |
| Q1 |  | 1 [Reference] |
| Q2 |  | 0.77 (0.63-0.93) |
| Q3 |  | 0.56 (0.38-0.82) |
| Q4 |  | 0.88 (0.57-1.36) |
| *P* for Trend |  | 0.640 |
| **Female, n = 7918** |  |  |
| Q1 |  | 1 [Reference] |
| Q2 |  | 0.91 (0.64-1.29) |
| Q3 |  | 0.42 (0.21-0.83) |
| Q4 |  | 1.01 (0.46-2.22) |
| *P* for Trend |  | 0.178 |

According to the interquartile range of normalized difference vegetation index (NDVI) exposure levels from low to high, the participants were divided into Q1, Q2, Q3 and Q4 groups. (N=26 482)

^a^Cox model was adjusted for age at the diagnosis date, occupation, county-level migrant population, drug resistance, annual average PM_2.5_ concentration, nighttime light, distance to the nearest roads, road length and road density.

**Table S6** **Adjusted hazard ratios (HRs) of the retreatment of tuberculosis with greenness exposure (500 m buffers around residential addresses) according to occupation.**

|  |  | **Model^a^** |
| --- | --- | --- |
| **Exposure （NDVI, 500-m buffer）** |  | **Fully adjusted HR (95% CI)** |
| **Agriculture, n = 15 561** |  |  |
| Q1 |  | 1 [Reference] |
| Q2 |  | 0.83 (0.61-1.14) |
| Q3 |  | 0.85 (0.58-1.25) |
| Q4 |  | 1.22 (0.81-1.84) |
| *P* for Trend |  | 0.001 |
| **Industry, n = 1209** |  |  |
| Q1 |  | 1 [Reference] |
| Q2 |  | 0.88 (0.46-1.69) |
| Q3 |  | 0.72 (0.23-2.28) |
| Q4 |  | 1.24 (0.15-10.38) |
| *P* for Trend |  | 0.552 |
| **Government, education, & retired, n = 3243** |  |  |
| Q1 |  | 1 [Reference] |
| Q2 |  | 0.95 (0.68-1.34) |
| Q3 |  | 0.73 (0.50-1.05) |
| Q4 |  | 0.46 (0.24-0.89) |
| *P* for Trend |  | 0.015 |
| **Others, n = 6469** |  |  |
| Q1 |  | 1 [Reference] |
| Q2 |  | 0.86 (0.6-1.13) |
| Q3 |  | 0.67 (0.48-0.93) |
| Q4 |  | 0.54 (0.25-1.15) |
| *P* for Trend |  | 0.015 |

According to the interquartile range of normalized difference vegetation index (NDVI) exposure levels from low to high, the participants were divided into Q1, Q2, Q3 and Q4 groups. (N=26 482)

^a^Cox model was adjusted for age at the diagnosis date, sex, county-level migrant population, drug resistance, annual average PM_2.5_ concentration, nighttime light, distance to the nearest roads, road length and road density.

**Table S7** **Adjusted hazard ratios (HRs) of the retreatment of tuberculosis with greenness exposure (500 m buffers around residential addresses) according to** **drug-resistant.**

|  |  | **Model^a^** |
| --- | --- | --- |
| **Exposure （NDVI, 500-m buffer）** |  | **Fully adjusted HR (95% CI)** |
| **Drug-resistant, n = 420** |  |  |
| Q1 |  | 1 [Reference] |
| Q2 |  | 0.79 (0.47-1.35) |
| Q3 |  | 0.72 (0.33-1.55) |
| Q4 |  | 0.83 (0.27-2.56) |
| *P* for Trend |  | 0.668 |
| **drug-sensitive, n = 26 062** |  |  |
| Q1 |  | 1 [Reference] |
| Q2 |  | 0.73 (0.61-0.87) |
| Q3 |  | 0.51 (0.36-0.72) |
| Q4 |  | 0.88 (0.60-1.29) |
| *P* for Trend |  | 0.002 |

According to the interquartile range of normalized difference vegetation index (NDVI) exposure levels from low to high, the participants were divided into Q1, Q2, Q3 and Q4 groups. (N=26 482)

^a^Cox model was adjusted for age at the diagnosis date, sex, occupation, county-level migrant population, annual average PM_2.5_ concentration, nighttime light, distance to the nearest roads, road length and road density.

**Table S8** **Adjusted hazard ratios (HRs) of the retreatment of tuberculosis with greenness exposure (500 m buffers around residential addresses) according to PM_2.5_ concentration.**

|  |  | **Model^a^** |
| --- | --- | --- |
| **Exposure （PM_2.5_）** |  | **Fully adjusted HR (95% CI)** |
| **Low^b^, n = 13 250** |  |  |
| Q1 |  | 1 [Reference] |
| Q2 |  | 0.66 (0.46-0.94) |
| Q3 |  | 1.13 (0.78-1.64) |
| Q4 |  | 1.34 (0.90-1.99) |
| *P* for Trend |  | <0.001 |
| **High^b^, n = 13 232** |  |  |
| Q1 |  | 1 [Reference] |
| Q2 |  | 0.82 (0.68-0.99) |
| Q3 |  | 0.73 (0.59-0.90) |
| Q4 |  | 0.79 (0.53-1.17) |
| *P* for Trend |  | <0.001 |

According to the interquartile range of normalized difference vegetation index (NDVI) exposure levels from low to high, the participants were divided into Q1, Q2, Q3 and Q4 groups. (N=26 482)

^a^Cox model was adjusted for sex, occupation, county-level migrant population, drug resistance, annual average PM_2.5_ concentration, nighttime light, distance to the nearest roads, road length and road density.

^b^PM_2.5_ represents for air pollution level with a median value of 79.54 μg/m^3^ as the low-high cutoff.

**Table S9** **Adjusted odds ratios (ORs) of the drug-resistant with greenness exposure (500 m buffers around residential addresses).**

|  |  | **Model^a^** |  | **Model^b^** |
| --- | --- | --- | --- | --- |
| **Exposure （NDVI, 500-m buffer）** |  | **Age-adjusted OR (95% CI)** |  | **Fully adjusted OR (95% CI)** |
| **All participants, n = 26 482** |  |  |  |  |
| Q1 |  | 1 [Reference] |  | 1 [Reference] |
| Q2 |  | 0.75 (0.59-0.95) |  | 0.99 (0.74-1.32) |
| Q3 |  | 0.40 (0.30-0.54) |  | 0.93 (0.52-1.65) |
| Q4 |  | 0.42 (0.31-0.56) |  | 0.93 (0.45-1.94) |
| *P* for Trend |  | <0.001 |  | 0.864 |
| **Tuberculosis retreatment, n = 1542** |  |  |  |  |
| Q1 |  | 1 [Reference] |  | 1 [Reference] |
| Q2 |  | 1.14 (0.69-1.87) |  | 1.24 (0.71-2.14) |
| Q3 |  | 1.03 (0.61-1.74) |  | 1.52 (0.58-3.99) |
| Q4 |  | 0.57 (0.31-1.05) |  | 0.91 (0.23-3.63) |
| *P* for Trend |  | 0.675 |  | 0.675 |

According to the interquartile range of normalized difference vegetation index (NDVI) exposure levels from low to high, the participants were divided into Q1, Q2, Q3 and Q4 groups. (N=26 482)

^a^Logistic regression model was adjusted for age at the diagnosis date.

^b^Logistic regression model was adjusted for age at the diagnosis date, sex, occupation, county-level migrant population, annual average PM_2.5_ concentration, nighttime light, distance to the nearest roads, road length and road density

**Abbreviations**

**HRs:** hazard ratios

**MDR-TB:** multidrug-resistant tuberculosis

**NDVI:** the normalized difference vegetation index

**NTL:** nighttime light

**ORs:** odds ratios

**PM_2.5_:** fine particulate matter with an aerodynamic diameter of 2.5 μm or less

**PTB:** pulmonary tuberculosis

**SDs:** standard deviations

**TB:** tuberculosis
